# Feature extraction method of EEG based on wavelet packet reconstruction and deep learning model of VR motion sickness feature classification and prediction

**DOI:** 10.1371/journal.pone.0305733

**Published:** 2024-07-19

**Authors:** Shuhang Luo, Peng Ren, Jiawei Wu, Xiang Wu, Xiao Zhang

**Affiliations:** 1 School of Medical Information and Engineering, Xuzhou Medical University, Xuzhou, China; 2 Engineering Research Center of Medical and Health Sensing Technology, Xuzhou Medical University, Xuzhou, China; National University of Sciences and Technology, PAKISTAN

## Abstract

The surging popularity of virtual reality (VR) technology raises concerns about VR-induced motion sickness, linked to discomfort and nausea in simulated environments. Our method involves in-depth analysis of EEG data and user feedback to train a sophisticated deep learning model, utilizing an enhanced GRU network for identifying motion sickness patterns. Following comprehensive data pre-processing and feature engineering to ensure input accuracy, a deep learning model is trained using supervised and unsupervised techniques for classifying and predicting motion sickness severity. Rigorous training and validation procedures confirm the model’s robustness across diverse scenarios. Research results affirm our deep learning model’s 84.9% accuracy in classifying and predicting VR-induced motion sickness, surpassing existing models. This information is vital for improving the VR experience and advancing VR technology.

## 1. Introduction

### 1.1 Background

Virtual reality (VR) has been a presence for many years, serving various domains [1]. Across these domains, VR video content spans applications in gaming, sports, education, travel guides, and clinical settings [2]. Despite its wide-ranging applications, VR video content carries potential adverse effects, such as Internet addiction [3], which has emerged as a primary concern for both users and content creators. Vision-induced motion sickness (VIMS), also known as virtual reality disease or VR disease, encompasses symptoms arising from exposure to virtual environments.

Manifesting as sweating, yawning, dizziness, spatial disorientation, instability, fatigue, nausea, and even vomiting [4, 5], virtual reality disease constitutes a form of discomfort associated with immersive environments. Researchers have explored various avenues to elucidate this phenomenon, investigating sensory conflicts, transmission delays, optical flow speed, background complexity, realism, and other contributing factors [6]. However, a comprehensive understanding of the principal neural mechanisms underlying these discomforts remains elusive.

### 1.2. Detection of individual motion sickness level

Developing quantitative methods for measuring virtual reality-induced ailments holds significant importance due to the limited availability of such methods. Presently, assessments predominantly rely on subjective measurements. The Virtual Reality Disease Susceptibility Questionnaire (MSSQ) [7] and Simulated Disease Questionnaire (SSQ) stand as commonly employed tools for qualitative assessment [8]. Additionally, the Fast Motion Sickness questionnaire (FMS) offers an improved means, of gathering subjective feedback through post-VR experience surveys to gauge motion sickness levels [9]. Despite their prevalent use, these questionnaires retain subjectivity and exhibit unreliability.

Some researchers have turned to sensor-based approaches involving accelerometers, gyroscopes, and magnetometers to track users’ head movements, tilts, and rotations [10]. Deviations or rapid movements of the head are thought to correlate with motion sickness occurrences [11]. Leveraging the human body’s diverse signals—such as electroencephalogram (EEG), electrocardiogram (ECG), electrogastrogram (EGG), heart rate variability (HRV), and galvanic skin response (GSR)—offers a means for quantitative and objective evaluation of reactions induced by virtual reality, including virtual reality-induced ailments [12–15]. Among these, EEG stands out as the most extensively employed in virtual reality research [16], serving purposes such as comparing the real and virtual world, cognitive training tasks, 2D and 3D display comparisons, virtual reality driving simulations, and more.

### 1.3 Application of EEG feature extraction and matching

Patients experiencing virtual reality-induced motion sickness exhibit heightened EEG activity in the frontal, temporal, and occipital regions compared to individuals without such symptoms [17]. Notably, augmented prefrontal cortex activity in these patients indicates a compromised cognitive control ability [18, 19]. Furthermore, reduced connectivity between prefrontal cortex regions in these individuals contributes to impaired information processing and compromised cognitive control [20]. Weakened activity in the visual cortex of these patients leads to disjointed visual information processing. Additionally, heightened temporal cortex activity may stem from a mismatch between the vestibular and visual systems among those with virtual reality-induced motion sickness [21]. The evaluation of human physiological states often leverages EEG signals due to their rich information directly linked to these states.

In processing EEG signals, aside from time-frequency domain features, spatial characteristics, coherence, and phase synchronization features are frequently employed. Advancements in deep learning and neural networks have introduced methods utilizing convolutional neural networks (CNN) and recurrent neural networks (RNN) for EEG feature extraction and classification [22–24]. Notably, wavelet transform feature decomposition has significantly progressed in EEG signal processing applications, enhancing comprehension of EEG signal characteristics and brain functions [25].

### 1.4 Application of wavelet transform feature decomposition

Certainly, researchers have employed various wavelet packet transforms along with statistical measurements to analyze EEG signals. Their findings indicate the effectiveness of wavelet packet decomposition in extracting features relevant to epilepsy. Studies have also integrated wavelet packet decomposition with brain network analysis to classify EEG signals [26]. These investigations highlight that wavelet packet feature decomposition can effectively extract pertinent features from EEG signals, enabling the classification of different brain states and offering support for diagnosing and monitoring brain disorders. Notably, wavelet packet feature decomposition aids in the early detection and classification of cognitive impairment when analyzing EEG signals [27].

As a result, wavelet packet features serve as a valuable tool for identifying abnormal patterns within EEG signals, with potential applications in predicting and assessing various early-stage diseases. Consequently, in this paper, we opt to utilize this method to process EEG signals, leveraging its efficacy in uncovering significant patterns and aiding in disease prediction and assessment.

### 1.5 Contributions and structure of this study

This paper aims to introduce a deep learning-based model capable of distinguishing between normal EEG states and states indicative of motion sickness. Furthermore, the model aims to predict the severity of motion sickness in real time by analyzing EEG signals, enabling early detection of motion sickness onset. The study’s primary contributions are outlined below:

1. Proposal of a novel approach for EEG feature extraction based on wavelet packet transform and reconstruction. This method amplifies the original EEG signal’s vector dimensionality and extracts signal features from individual frequency bands.
2. Introduction of a circular neural network model within the domain of deep learning. This model integrates classification and prediction functionalities, allowing accurate classification and prediction of motion sickness states inferred from EEG signal analysis.
3. Rigorous evaluation of the model’s performance using benchmark datasets across various EEG data classification tasks of differing temporal extents and prediction tasks employing diverse window sizes. This evaluation enables the identification of optimal parameters related to temporal length and window dimensions. Comparative analysis against alternative machine learning models demonstrates a noticeable enhancement in the proposed model’s predictive abilities.

The experimental results showcase a strong correlation between the proposed model’s metrics and subjective scores. Notably, the proposed method exhibits a substantial improvement of approximately 19% in the Prediction Likelihood Correlation Coefficient (PLCC) compared to assessments based solely on physiological signals such as heart rate and GSR.

However, it is difficult to find a study to evaluate the correlation between EEG and Virtual Reality Disease Questionnaire (SSQ). Therefore, in this paper, we propose an alternative method to predict the user’s motion sickness level in a virtual reality environment by using EEG and benchmark based on subjective scores. The second section introduces our method, including EEG feature extraction, and proposes a framework for the deep learning model. The third section introduces data and experimental settings. The fourth section discusses the results of experiments and performance evaluation. The fifth section is the conclusion, which summarizes the advantages of the model and the limitations of the research.

## 2. Dataset and methods

### 2.1 Dataset

In this section, we will thoroughly outline the data collection process involving 25 subjects, delve into the preprocessing steps applied to EEG signals, and explain the dataset division for experiments with deep learning models. The detailed exploration covers the methodologies used to gather diverse and representative data from a subject pool.

Post-data acquisition, we shift focus to the preprocessing of EEG signals, detailing the steps taken to clean, filter, and enhance the data. This phase is crucial for ensuring data quality and reliability, contributing to the robustness of subsequent analyses. Additionally, we elaborate on the dataset division for experiments involving deep learning models, explaining the rationale behind dividing the dataset into training, validation, and testing sets. This systematic division is vital for training and evaluating the performance of our models in an unbiased manner. By elucidating the collection process, preprocessing techniques, and dataset division strategies, this chapter establishes a comprehensive foundation for understanding the data-related aspects of our research, ensuring the integrity of data used in subsequent experiments.

#### 2.1.1 Dataset description

**Fig 1:**
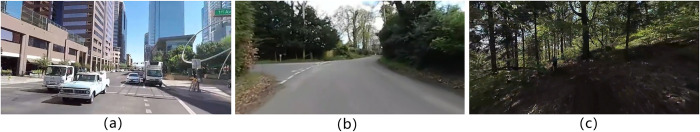
The experimenter wears a head-mounted display to watch the virtual display scene. (a) Urban road driving; (b) Small town road riding; (c) Jungle cycling.

The data acquisition equipment uses HTC VIVE Pro Eye, a head-mounted display with VR technology, which users can wear to watch high-quality VR video content. Our collected data include the electroencephalogram (EEG) data collected by 25 subjects after watching the video data composed of 20 videos and the corresponding personal subjective SSQ score. Approved by the Medical Ethics Committee of the Affiliated Hospital of Xuzhou Medical University, the trial number is XFY 2022-KL 430–01. On 1 March 2023, the preliminary recruitment of subjects was started at Xuzhou Medical University, and the recruitment was completed in about one week. After the recruitment, the subjects were informed about some precautions and informed consent, and the collection time of each subject was arranged. Then, the start date of the experiment was 8 March 2023, and the end date of the experiment was 30 March 2023.All subjects signed the informed consent form before the experiment. Before the experiment, the subjects will be informed of all the precautions and sign the written informed consent form, and arrange the collection time for each subject. Then the experiment began on March 8, 2023 and was completed before March 30, 2023. All subjects signed the informed consent form before the experiment. The subject’s vision is normal or corrected to normal. In our experiment, before watching each content, they sit in a rotatable chair from zero position, so as to freely watch the 360-degree content. To investigate the effect of various motion patterns on VR sickness, they collected 360-degree video datasets with various motion patterns from static to dynamic for these subjects. The video database contains driving, flying, roller coaster, riding, and other scenes. We list three VR scenes in Fig 1: urban road driving, small-town riding, and jungle cross-country.

The 16-item SSQ consists of 16 physical symptoms, which are closely related to VR sickness, with a discrete four-point grading scale for each symptom (0: None, 1: Slight, 2: Moderate, 3: Severe). 16 physical symptoms are used to evaluate the level of motion sickness, where x, y and z represent the Score. Also, the indicators are assigned weights (9.54, 7.58, 13.92) respectively to calculate three separate symptoms (Nausea, Oculomotor and Disorientation). The scores of each subject after watching each video were averaged to eliminate the specificity of individual differences in scoring.

The formula used to calculate the total score is expressed as:

$$\begin{array}{l} SSQ_{\mathrm{total}}=3.74\times (\;\frac{1}{9.54}SSQ_{\mathrm{Nausea}}+\frac{1}{7.58}\\ \;\times SSQ_{\mathrm{oculo}}+\frac{1}{13.92}SSQ_{\mathrm{Dis}}). \end{array}$$ (1)

**Fig 2:**
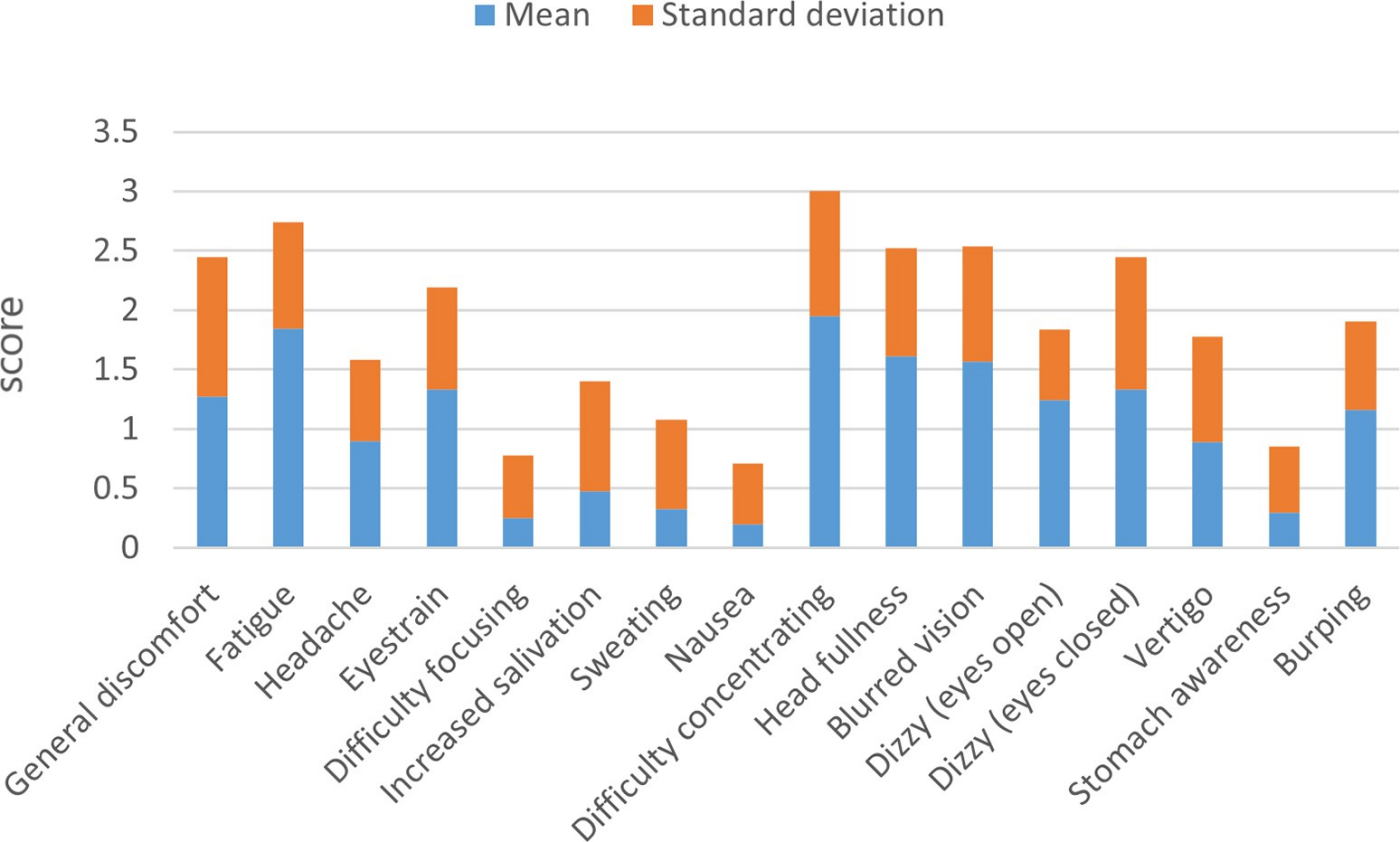
The average and standard deviation of 16 individual symptoms of 25 subjects in the questionnaire.

In Fig 2, we evaluated the participants’ VR motion sickness in the virtual reality (VR) environment in many dimensions, and measured the average feeling level and the change degree of feeling degree in each evaluation dimension, and presented the research results through the statistical indicators of average value and standard deviation. According to the statistical data, the average uncomfortable feeling reported by participants when experiencing virtual reality is between 1.28 and 1.96, including a series of feelings, such as general discomfort, fatigue, headache, eye fatigue, difficulty in concentration and so on. The calculation results of standard deviation show the degree of variation between these feelings, some of which have large standard deviations, indicating that participants have great individual differences in their feelings, while some have small standard deviations, indicating that participants have relatively consistent feelings for these feelings. These data provide a basis for further analysis of individual differences in uncomfortable feelings in virtual reality experience.

**Fig 3:**
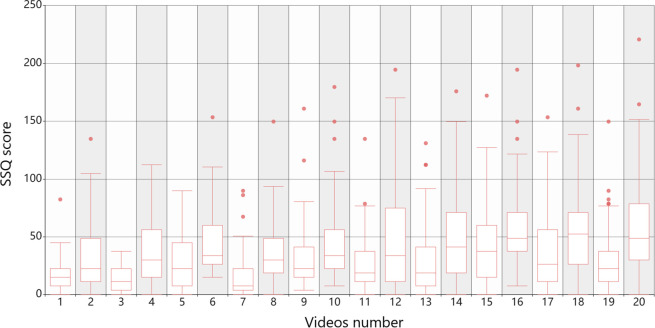
25 subjects watched SSQ score box chart of 20 videos.

As shown in Fig 3 below, we counted the SSQ scores collected in each video scene into a box chart. From the statistical data, most experimenters had different degrees of motion sickness during the collection, and recorded this state by using the supervisor questionnaire, to map the differences of EEG signals to the scores. It can be seen that in different tasks, most subjects have different degrees of motion sickness, and some subjects even reach more than 100, which shows that the selected task scene can meet the conditions for making the subjects reach motion sickness. In this state, EEG signals are the key data for us to extract features. The reason why there are outliers in the picture is that some experimental subjects watching videos have different levels of motion sickness tolerance and the complexity and uncertainty of the scenes they watch, which makes their SSQ scores much higher than the average, which is a normal phenomenon. The EEG data of these experimental subjects will not be excluded from being input into the model to eliminate the influence of the prediction model on individual differences and increase the stability and performance of the model.

#### 2.1.2 EEG signal acquisition and preprocessing

Cognionics AIM is used to collect EEG signals at a frequency of 500hz. The EEG data in the normal state and motion sickness state were collected for two minutes, and the EEG data contained 32 channels. According to the conclusions of previous research literature on motion sickness, VR motion sickness occurs in the parietal lobe, occipital lobe, frontal lobe and temporal lobe. To avoid too many channels of EEG data and irrelevant channels reducing the effect of feature extraction, according to the relationship between brain regions and EEG signal acquisition channels, we select the corresponding EEG channels for processing, and we only select eight channels as follows: FP1, FP2, C3, C4, P3, P4, O1 and O2 channel.

**Fig 4:**
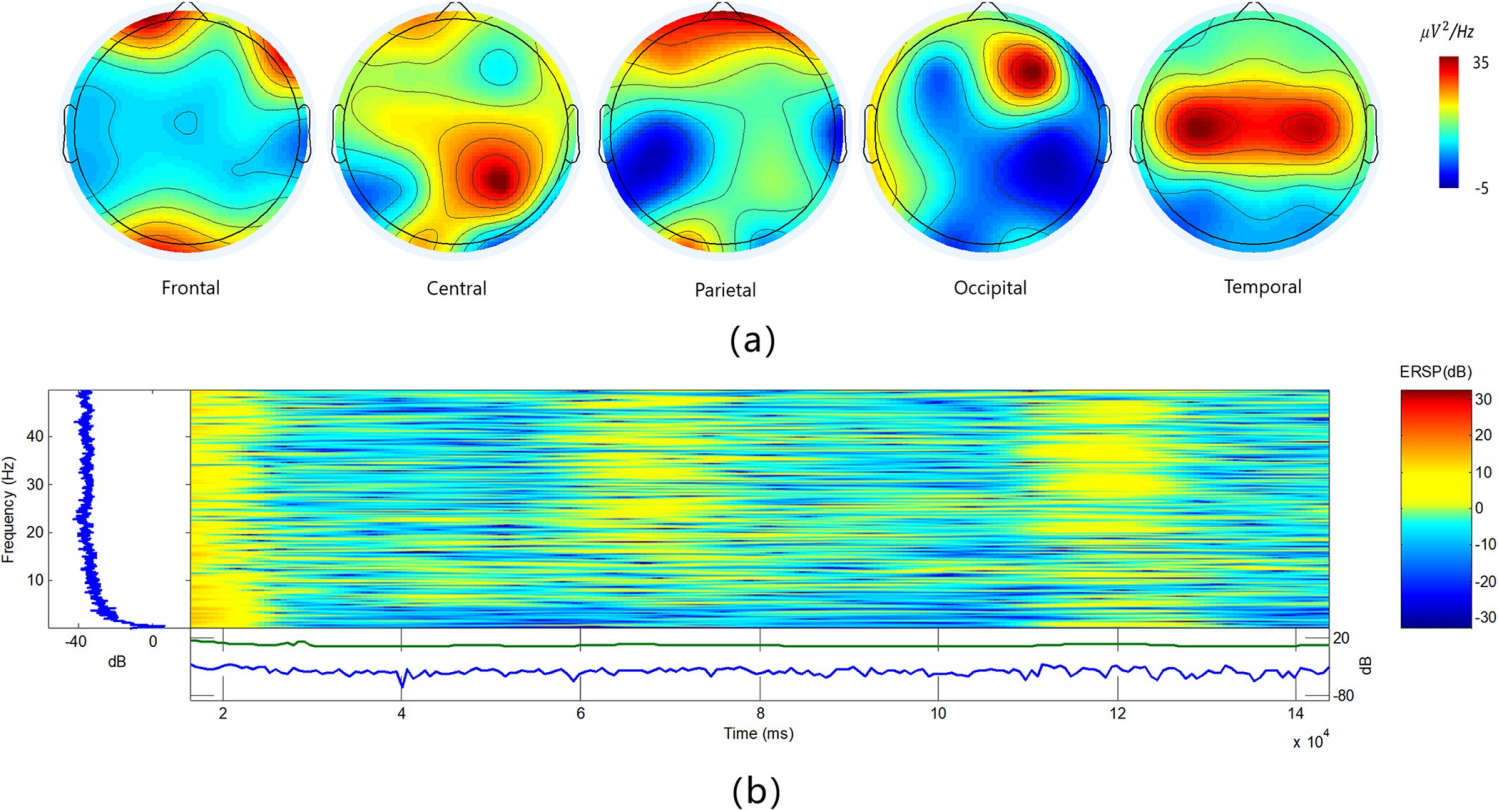
The time-frequency domain analysis of topographic maps of five brain regions is carried out by EEGLAB v13.0. Part (a) is the topographic map of brain area, and part (b) is the time-frequency domain drawn by short-time Fourier transform when motion sickness occurs.

Given that the dataset under consideration is in its raw, unprocessed state, it necessitates a series of meticulous preprocessing steps to render it amenable for subsequent analysis. The following methodological procedures are meticulously adhered to in this data preparation process:

To commence, the initial step involves filtering the EEG signal, whereby a bandpass filter in the range of 0.1-100Hz is applied, with a sampling rate of 500Hz meticulously adhered to. This crucial stage ensures that the signal is conditioned to contain pertinent frequency components, essential for the ensuing analysis. Subsequently, the EEG data is imported into EEGLAB version 13.0.0, and a sophisticated band-pass filtering operation is employed. A precise band-pass filter encompassing the range of 0.5–50 Hz is applied, refining the data for further analysis [28]. Simultaneously, electrode localization information is imported, which facilitates the identification and removal of superfluous electrodes. In alignment with the requirements of the classification task, electrodes that do not contribute substantively are diligently omitted. Moreover, for the purpose of segmentation, electrodes that extend beyond the stipulated classification task length are judiciously excised, ensuring that the dataset is optimized for the subsequent processing stages. The rectification of artifacts is another critical step in data preprocessing. Principal Component Analysis (PCA) is employed to detect and eliminate any unwanted artifacts, which could potentially confound the subsequent analysis. Furthermore, the data is carefully re-referenced using the bilateral mastoid reference points [29]. This re-referencing step is instrumental in ensuring that the EEG data is suitably configured for the subsequent application of wavelet packet transform techniques. In Fig 4, By using EEGLAB for analysis, we deeply studied the frequency transformation and time-frequency domain of each brain region when motion sickness occurred. The purpose of this analysis is to reveal the dynamic changes of EEG signals during uncomfortable experiences, to understand the neural mechanism of motion sickness more comprehensively. Through the detailed exploration of frequency transformation, we can identify the activity level of specific frequency bands in different brain regions, thus revealing the influence of motion sickness on EEG activity. Time-frequency domain analysis provides a more detailed understanding of the changes of EEG signals in time and frequency, which helps capture the time sequence characteristics of motion sickness events. When motion sickness occurs, the EEG signals in the Frontal, Central, Parietal, Occipital and Temporal areas will change, such as the frequency activities in the Frontal and Temporal areas will increase, the Alpha in Central and Occipital areas will decrease, and the Beta and Gamma band activities will increase. Except for the Beta band activities, the Parietal area is the same as the Central and Occipital areas.

These intricate preprocessing measures are imperative in preparing the raw EEG data for the forthcoming wavelet packet transform analysis, ensuring that the dataset is optimized for precise and meaningful insights in our research endeavor.

#### 2.1.3 Classification and prediction training set

To achieve the best performance in motion sickness feature recognition for the classification task, careful consideration of window width is paramount. A balance between overloaded information and inadequate feature extraction within EEG signals is crucial. This study adopts fixed window widths—5, 10, and 12 seconds—aligning with prior research suggesting 3–12 seconds as an optimal interval for emotional state identification using EEG signals [30, 31]. Consequently, three distinct sets of EEG samples were obtained, totaling 4800, 2400, and 2000 samples, respectively, corresponding to the designated window widths. The classifier’s effectiveness in categorizing these samples guides the selection of the optimal window width, ensuring precise and meaningful analysis.

In contrast, the predictor, tasked with regression using extended EEG segments, circumvents data segmentation. Instead, it harnesses the entirety of questionnaire data and unaltered continuous EEG signals for input. This holistic approach empowers the predictor to learn motion sickness characteristics through comprehensive time series network-based feature extraction and recognition, enabling real-time prediction of motion sickness levels with precision.

The experimental evaluation was conducted in a controlled academic laboratory environment using a widely recognized benchmark database. A computational setup comprising an Intel Core i5-11400H processor, 16 gigabytes of memory, and an NVIDIA RTX 3050 TI graphics card facilitated model implementation using the Keras framework.

For model pre-training, original EEG data from KAIST IVY LAB were selected and processed [32]. The learner underwent 50 epochs of pre-training using the ADAM optimizer [33] with a batch size of 4, setting the initial learning rate at 0.00005 and incorporating β1 and β2 values of 0.9 and 0.999, respectively. A weight decay of {10}^{−8} was applied in each iteration.

Notably, both the classifier and predictor underwent training under identical optimizer settings, ensuring fairness and consistency in evaluating their performances. This uniformity in training conditions is critical for unbiased assessment.

### 2.2 Methods

**Fig 5:**
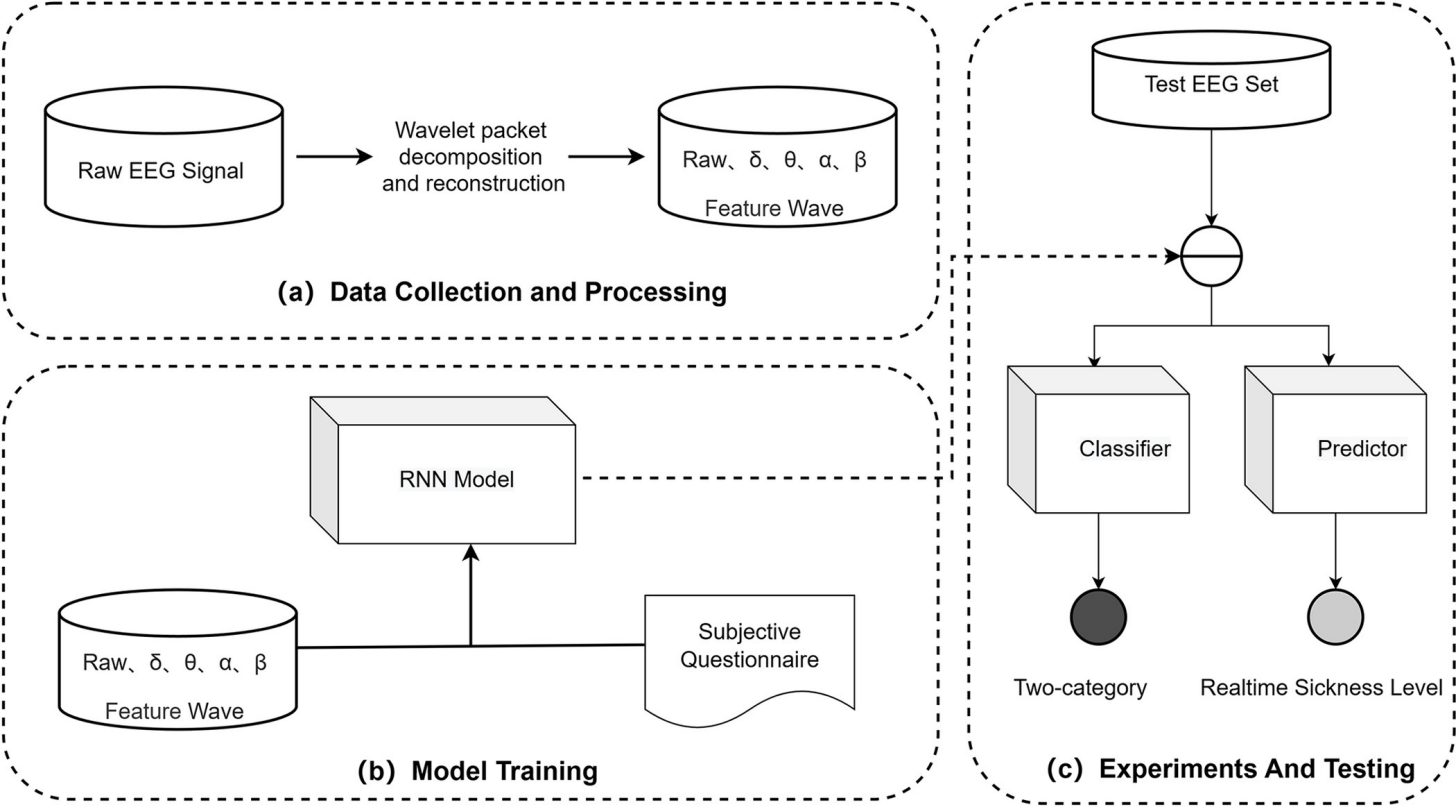
Overall process of the proposed model framework. (a) Data Collection and Processing: Primarily, meticulous acquisition and processing of EEG signals and questionnaire data lay the foundation for this method. Raw data undergoes a transformational journey to become structured feature vectors conducive to computational analysis. Preprocessing involves the application of the wavelet packet transform [34] to EEG data, stratifying it into δ, θ, α, and β frequency components. This nuanced stratification aids in comprehending evolving EEG features over time, enhancing the model’s predictive accuracy. (b) Model Training: The training phase involves feeding preprocessed EEG signals and questionnaire data into the model’s architecture. A Recurrent Neural Network (RNN) takes charge, extracting distinct features characterizing different states. These features, essential for subsequent modeling, are then mapped to questionnaire responses, enabling the model to discern motion sickness states. (c) Test Data Evaluation: The final step involves rigorous evaluation using a classifier and predictor to assess the model’s performance on test data. This phase offers insights into the model’s generalization ability and predictive accuracy.

This chapter details the methodology for classifying and real-time predicting motion sickness states within a VR environment by leveraging EEG signal characteristics through a deep learning model. The schematic representation in Fig 5 delineates the construction process of this method.

These fundamental processes—data collection and processing, model training, and test data evaluation—drive the proposed model’s ability to decipher EEG signal intricacies and comprehend cognitive states. Ultimately, the model can discern information differences between normal and motion sickness states, facilitating real-time prediction of motion sickness levels within unfamiliar EEG signals.

#### 2.2.1 Wavelet packet decomposition and reconstruction

It is mentioned in the introduction that the methods of feature extraction of EEG signals are different in various fields, and the effect of feature extraction needs to be determined according to the situation. Because we try to predict EEG signals, we need to analyze them in the time and frequency domain. The wavelet packet transform has many advantages in this respect, which will be mentioned later, so we use this method to extract the features of EEG.

Wavelet analysis and wavelet packet analysis are suitable for non-stationary signal analysis. Compared with wavelet analysis, wavelet packet analysis can be used to analyze the signal more carefully, and wavelet packet analysis can divide the time-frequency plane more carefully [35, 36], and the resolution of the high-frequency part of the signal is better than that of wavelet analysis. According to the characteristics of the signal, the best wavelet basis function can be adaptively selected to analyze the signal better, so wavelet packet analysis is more widely used [37].

To input the signal characteristics of EEG signals in each frequency domain as feature vectors, we use the db4 wavelet base to decompose EEG signals into 8 layers of wavelet packets and reconstruct the frequency bands we need. Firstly, the original EEG data of 500hz are processed by wavelet tools in the MATLAB toolkit. A Wavelet packet is decomposed by a wavelet toolkit to form a wavelet packet tree.

A Packet represents approximate components and contains low-frequency information about the signal, while D packet represents detailed components and contains high-frequency information or details of signals. Discrete filters and down-sampling operations are commonly used [38]. hk\*left*(\*right*) and gk\*left*(\*right*) respectively represent the low-pass filter and the high-pass farer corresponding to the orthogonal scale function in the wavelet packet transform. The wavelet packet transforms coefficients of the original signal f\*left*(*t*\*right*) at the i−th\ node on the decomposition level j are as follows:

$$d_{j}^{2i}(t)=\sum_{k=-\infty }^{+\infty }h(k)d_{j-1}^{i}(2t-k),$$ (2)

$$d_{j}^{2i+1}(t)=\sum_{k=-\infty }^{+\infty }g(k)d_{j-1}^{i}(2t-k),$$ (3)

Where k is the translation parameter.

When we reach the last layer of the wavelet packet tree, we get the decomposed signal of each frequency band, and then use the same wavelet packet basis function and decomposition level to reconstruct the required frequency band through inverse wavelet packet decomposition. This process is the inverse operation of decomposition, which is used to recombine details and approximate coefficients into signals.

Wavelet packet transform has the characteristic of equal frequency band decomposition, and different nodes correspond to different EEG signal frequency bands. It should be noted that the order of wavelet packet nodes and the distribution order of frequency bands obtained by wavelet packet decomposition of EEG signals are staggered [39]. To reconstruct the frequency band, we need to get the input signals of δ, θ, α and β bands, which are used as the input of the model together with the original signals, forming the feature vector of the model extraction features.

**Fig 6:**
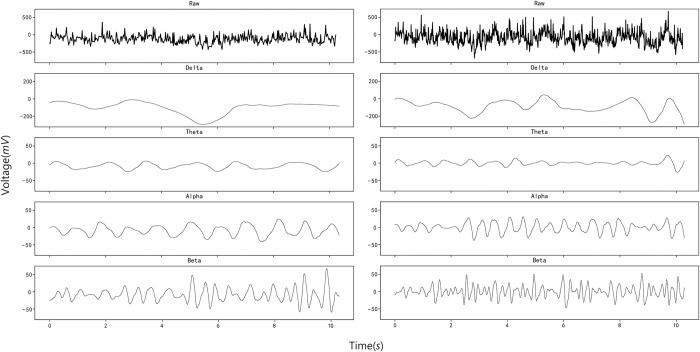
Characteristics of EEG in normal and motion sickness states.

As can be seen in Fig 6, the fluctuation of EEG signals in the normal state and motion sickness state is different in each frequency band, and it has rhythm. The purpose of feature extraction can be achieved by inputting this electric amplitude and rhythm into the circulatory neural network and learning different features of the two States. The left side of the figure shows the signal characteristics of a normal EEG signal with a length of 10s after wavelet packet transformation and reconstruction in the frequency band we need. On the right side of the figure are the signal characteristics of the same length EEG signal after the same processing in the motion sickness state. It can be seen that when motion sickness occurs, the δ, θ, α and β rhythms of EEG signals will fluctuate greatly, which is consistent with the conclusions obtained in previous studies. The result is that we extract the time-frequency domain features of EEG and input them into the model as amplified data, which greatly improves the accuracy and dimension of EEG feature recognition.

#### 2.2.2 Feature extraction model of EEG signal based on GRU

The selection of the Gated Recurrent Unit (GRU) network as the intermediary layer for characterizing EEG signals is underpinned by a host of compelling reasons, which collectively underscore its suitability for the task at hand.

To commence, it is imperative to recognize that GRU stands as a variant of the Recurrent Neural Network (RNN) architecture, renowned for its formidable prowess in sequence modeling. Given that electroencephalogram (EEG) signals inherently manifest as time series data, the imperative lies in capturing the nuanced temporal relationships that underpin these signals. GRU excels in this domain, adeptly unraveling the intricate time-dependent characteristics embedded within EEG signals.

Furthermore, the differentiating factor that sets GRU apart from its conventional RNN counterpart is the introduction of a gating mechanism [40]. This pivotal innovation empowers GRU to effectively address the conundrum of long-term dependence, a salient challenge in EEG signal analysis. As EEG signals may exhibit information propagation and evolution across protracted time spans, GRU’s capacity to mitigate this issue holds substantial relevance. Additionally, GRU distinguishes itself by its parsimonious parameterization, endowing it with an inherent efficiency in parameter learning. This not only expedites the model’s training process but also assumes added significance when dealing with voluminous EEG datasets.

**Fig 7:**
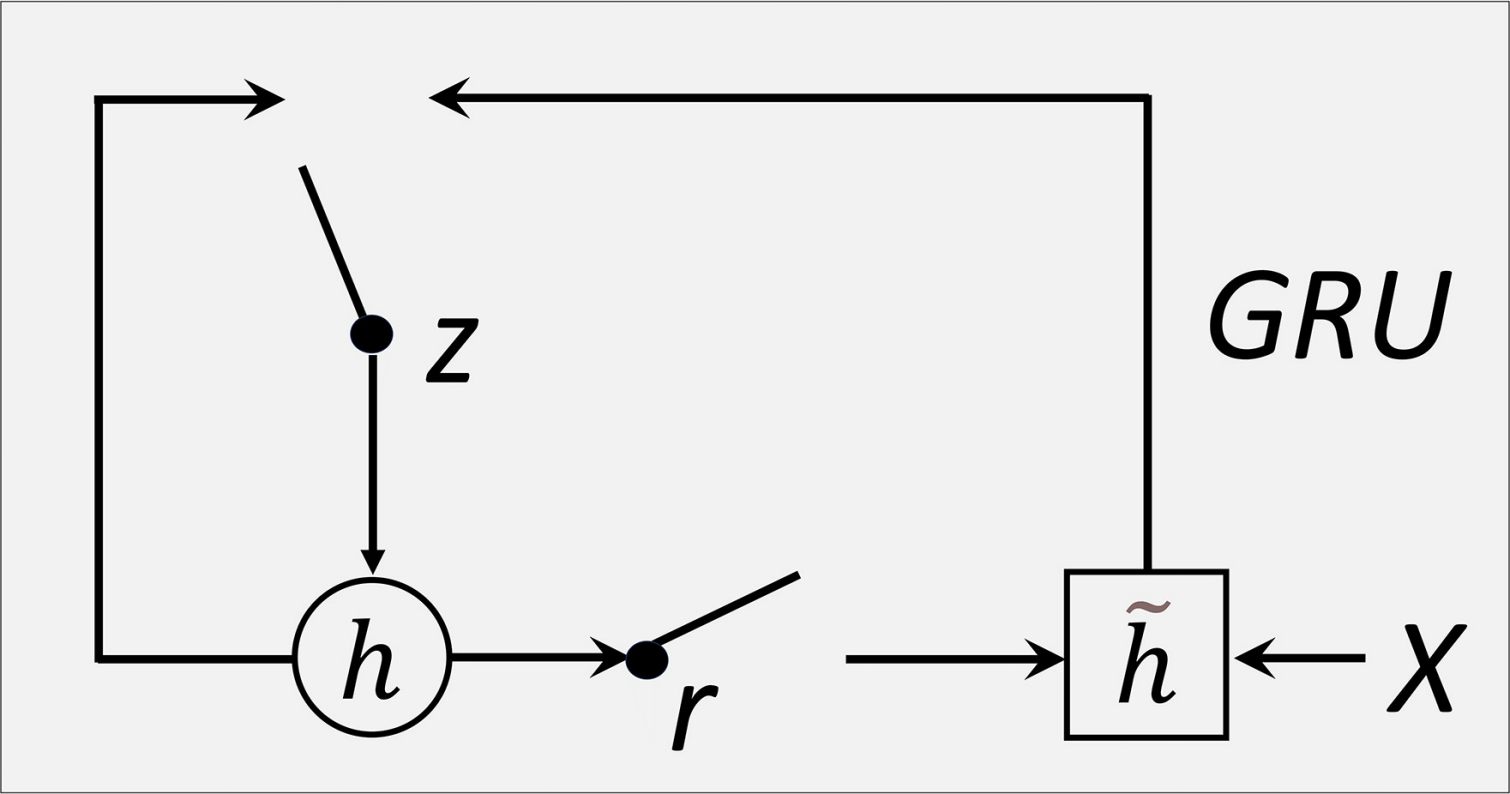
Structure of Gated Recurrent Unit.

Fig 7 shows the internal structure of the GRU model, including the update gate and reset gate. But it is an improvement of LSTM. It combines the forgetting gate and the input gate into an update gate, and at the same time, it combines the memory cell and the hidden layer into a reset gate, which further simplifies the operation of the whole structure and enhances its performance.

Update and control the hidden state at different time steps. GRU usually performs well in practice because it has fewer parameters and can handle long sequences of information. The model parameter transfer formula is as follows:

$$z_{t}\;=\sigma (W_{z}\cdot [h_{t-1},x_{t}]),$$ (4)

$$r_{t}\;=\sigma (W_{r}\cdot [h_{t-1},x_{t}]),$$ (5)

$${\tilde{h}}_{t}\;=\tanh\left(W\cdot [r_{t}*h_{t-1},x_{t}]\right),$$ (6)

$$h_{t}\;=(1-z_{t})*h_{t-1}+z_{t}*{\tilde{h}}_{t},$$ (7)

The network includes an update gate, and a reset gate, and the new hidden state calculated in time step t in the forward transmission process is controlled by the update gate *z*_*t*. If *z*_*t* is close to 1, most of the information of the old hidden state *h*_{*t*−1} will be preserved; if it is close to 0, the candidate hidden state *h*_*t* will be adopted.

The reset gate determines how to consider the past internal state. It controls which information should be forgotten or reset. The output of the reset gate is also in the range of (0, 1), with a value close to 0 indicating to forget the past internal state and a value close to 1 indicating to remember the past internal state. Then the output information at time t is *h*_*t* = {\*acute*{*h*}}_{*t*−1}+{\*widetilde*{*h*}}_*t*.

Input includes signal data in the time window and signal characteristics of each frequency spectrum. Using the data of the training set, the GRU model is trained by the backpropagation algorithm and Adam optimizer. During the training period, the model will try to learn the pattern of input EEG signal characteristics and the signal correlation relationship in the next time window. By minimizing the loss, this model is used to learn the characteristics of EEG signals with time series characteristics into the parameters of the model.

The feature extraction model will be combined with the classifier and predictor respectively to realize corresponding functions and the specific process will be introduced in the following sections.

In summary, the adoption of the GRU network as the intermediary layer within our model is judiciously grounded in its superlative aptitude for sequence modeling, adept handling of long-term dependence intricacies, efficient parameter learning, stable gradient propagation, and versatile applicability across diverse analytical tasks. The amalgamation of these attributes renders GRU a robust and adaptable tool for the precise characterization and comprehension of EEG signals, furthering the scope and sophistication of our research endeavors.

**Fig 8:**
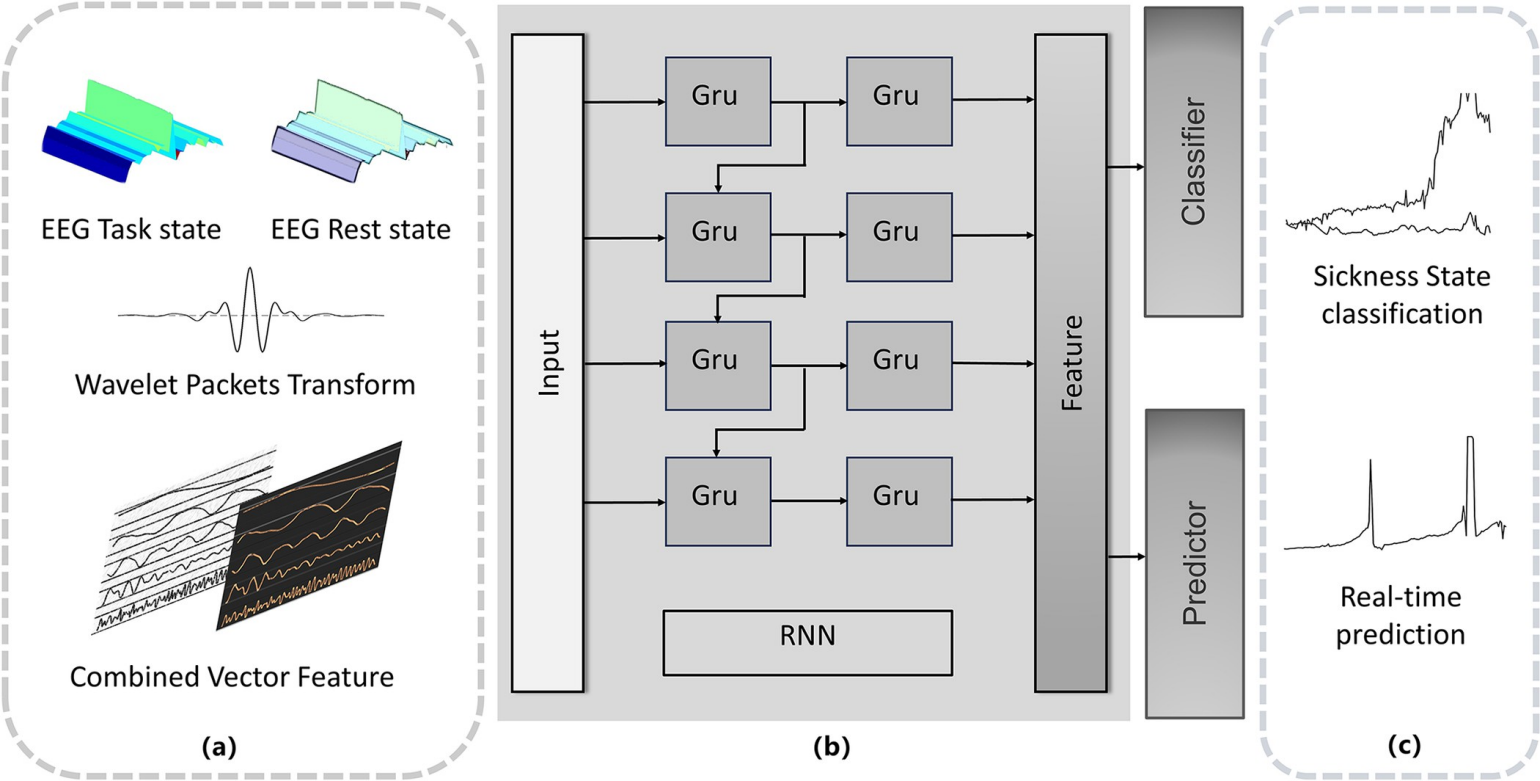
The framework of GRU-based learning classification and prediction model for learning EEG features is proposed.

The comprehensive process of constructing and training the entire model is meticulously illustrated in Fig 8, which encapsulates the essence of our methodology. The following subsections elucidate the intricacies of this procedure: (a) In the initial stage, the preprocessed EEG feature vectors are introduced into the Gated Recurrent Unit (GRU) model. This step serves as the foundation for learning and extracting the salient features embedded within the EEG feature vectors. (b) Subsequently, the cyclic neural network, as represented by the GRU model, actively engages in the process of feature acquisition. Each parameter of the network is subjected to a back-propagation mechanism, iteratively optimizing the model’s performance. Through the iterative minimization of loss, the parameters evolve, eventually converging to their final values, which constitute the learned model. (c) In the final phase, aligning with the specific task requirements, the test dataset is introduced into the model. This model includes classifiers and predictors meticulously trained to execute the tasks of EEG signal classification and prediction, catering to the specific objectives at hand.

#### 2.2.3 Classifier based on EEG characteristics

The classifier designed for discerning motion sickness-related features within EEG signals is pivotal in determining the presence of motion sickness states. Table 1 offers an in-depth view of the deep learning model’s architecture tailored specifically for this classification task.

**Table 1:** The architecture of classifier.

Layer	Type	Nodes	Activision function
**Input Layer**	Dense	40	Relu
**GRU Layer**	GRU	32	Tanh
**Dense Layer1**	Dense	16	Relu
**Dense Layer2**	Dense	8	Relu
**Output Layer**	Dense	1	Sigmod

The classifier comprises two discrete hidden layers, strategically structured to progressively extract and refine features. The initial hidden layer consists of 16 nodes, adept at intricate feature extraction, followed by a subsequent refinement to 8 nodes in the final hidden layer, consolidating the assimilated information.

The input layer is thoughtfully designed to accommodate a diverse array of nodes, encompassing representations of the original EEG data and brainwave frequency vectors (α, β, δ, and θ frequencies). This comprehensive input layer ensures the model can leverage a wide spectrum of pertinent information encoded within the EEG signals.

At the apex of this architecture lies the output layer, featuring a single node pivotal in determining the binary outcome: identifying the presence ("sickness") or absence ("normal") of motion sickness states. For binary classification tasks, the binary cross-entropy loss function [41] is paramount. Its selection is rooted in the model’s proficiency in handling binary classification problems, ensuring precise discrimination between desired outcomes.

Its loss function formula is:

Lcla(y,p)=−1n∑i=1Nyilog(p(yi))+(1−yi)log(1−p(yi)), (8)

y is the actual binary label (0 or 1), the normal state is 0 and the motion sickness state is 1.

In the initial phase of our methodology, the classified EEG data undergoes a rigorous training process, which is instrumental in discerning the nuances that characterize different states. This process hinges on the acquisition of different features, which subsequently serve as the input for the training classifier.

The crux of this training regimen revolves around the adept manipulation of the parameters embedded within the hidden layers. These parameters play a pivotal role in affecting the learning process, as they actively facilitate the comprehension of distinctions between the two states under investigation.

Upon the successful completion of this training endeavor, the culmination is marked by the attainment of a proficient and well-honed classifier. This classifier, having imbibed the intricacies of EEG data classification and the nuanced differences between states, stands poised to contribute significantly to the broader objectives of our research.

#### 2.2.4 Predictor based on EEG frequency band characteristics

After the classifier training, we can get a good ability of EEG classification task, so we should use the time sequence of features to predict the motion sickness level of real-time EEG signals.

A deep learning model architecture for predicting motion sickness from the user’s brain waves is shown. The input nodes are original data and brainwave frequency values with frequencies of α, β, δ and θ. Its characteristics are that as the input of the predictor, the predictor consists of four hidden layers, the first hidden layer consists of 32 nodes. The following two hidden layers, each endowed with 16 nodes, actively participate in the ongoing refinement process. The final hidden layer, comprising 8 nodes, takes on the mantle of synthesizing the information gleaned from the preceding layers. The ultimate layer in this architectural ensemble is the output layer, which plays a pivotal role in generating predictions. Its configuration ensures that the model is well-equipped to make insightful predictions regarding motion sickness.

Table 2 shows the framework of the deep learning model for EEG signal prediction of motion sickness. For the length of the prediction window, some studies have found that a longer window length can provide more historical information, which is helpful for the RNN model to better capture the long-term dependence of time series data [42]. This can improve the accuracy of forecasting, especially in tasks that require long-term memory, such as stock price forecasting or weather forecasting. However, if the window length is too long, the model may be too complicated, which may increase the calculation cost, and may not capture the short-term changes of data. This may reduce the prediction accuracy, especially for tasks that need to predict rapidly changing data [43].

**Table 2:** The architecture of predictor.

Layer	Type	Nodes	Activision function
**Input Layer**	Dense	40	Relu
**GRU Layer**	GRU	32	Tanh
**Dense Layer1**	Dense	32	Relu
**Dense Layer2**	Dense	16	Relu
**Dense Layer3**	Dense	16	Relu
**Dense Layer4**	Dense	8	Relu
**Output Layer**	Dense	1	Sigmod

To develop robust prediction models, we have thoughtfully employed distinct window lengths, specifically 5 seconds, 10 seconds, and 20 seconds, in the training process. This strategic choice enables the creation of three distinct prediction models, each fine-tuned to cater to the temporal nuances of the task at hand.

Ultimately, the determination of the optimal prediction window length for the model is contingent on its ability to effectively correlate with the real-time motion sickness state exhibited by the experimental subjects. This critical evaluation serves as the litmus test for the model’s efficacy and its alignment with the dynamic nature of motion sickness.

Throughout the training process, the GRU model plays a pivotal role. As it navigates the intricate neural network architecture, the model endeavors to map the disparities between the resting state and the task state to the extent represented by the Subjective Score of Sickness (SSQ). This calibrated mapping process is pivotal in enabling the model to make accurate predictions regarding the real-time motion sickness level, serving as a testament to its effectiveness and robustness in the context of our research. Finally, we judge the optimal prediction window length of the model by evaluating its correlation with the real-time motion sickness state of the experimental object. After two layers of dense net and output layer, in the training process, the GRU model maps the difference between the resting state and task state to the size of SSQ as much as possible, to predict the real motion sickness level.

The difference of EEG between normal state and motion sickness state (*E*_*t*) is calculated as:

$$E_{t}=|x_{t}-R_{\theta }(x_{t})|,$$ (9)

The loss function of the predicted value LP of VR disease score is expressed as:

Lpre=1N∑n=1N∥fGRU(Etn)−SSQtotaln∥2, (10)

Where N represents the number of batches and SS*Q*_{\*mathrm*{*total*\}}^*n* represents the true subjective score, which is defined as the total SSQ score of the *N-th* video sequence. *f*_{*GRU*} stands for predictor function.

## 3. Results and discussion

For the classification task, we compare the accuracy (Acc), specificity (SPE), and sensitivity (SEN) of three groups of 5s, 10s and 12s EEG data sets on the classifier to evaluate the performance of the classifier. For the regression task of the predictor, different prediction windows will also lead to different prediction performances. PLCC, SROCC and RMSE are used for evaluation, and the results are compared with those predicted by other algorithms on the benchmark database.

### 3.1 Classification task results

**Fig 9:**
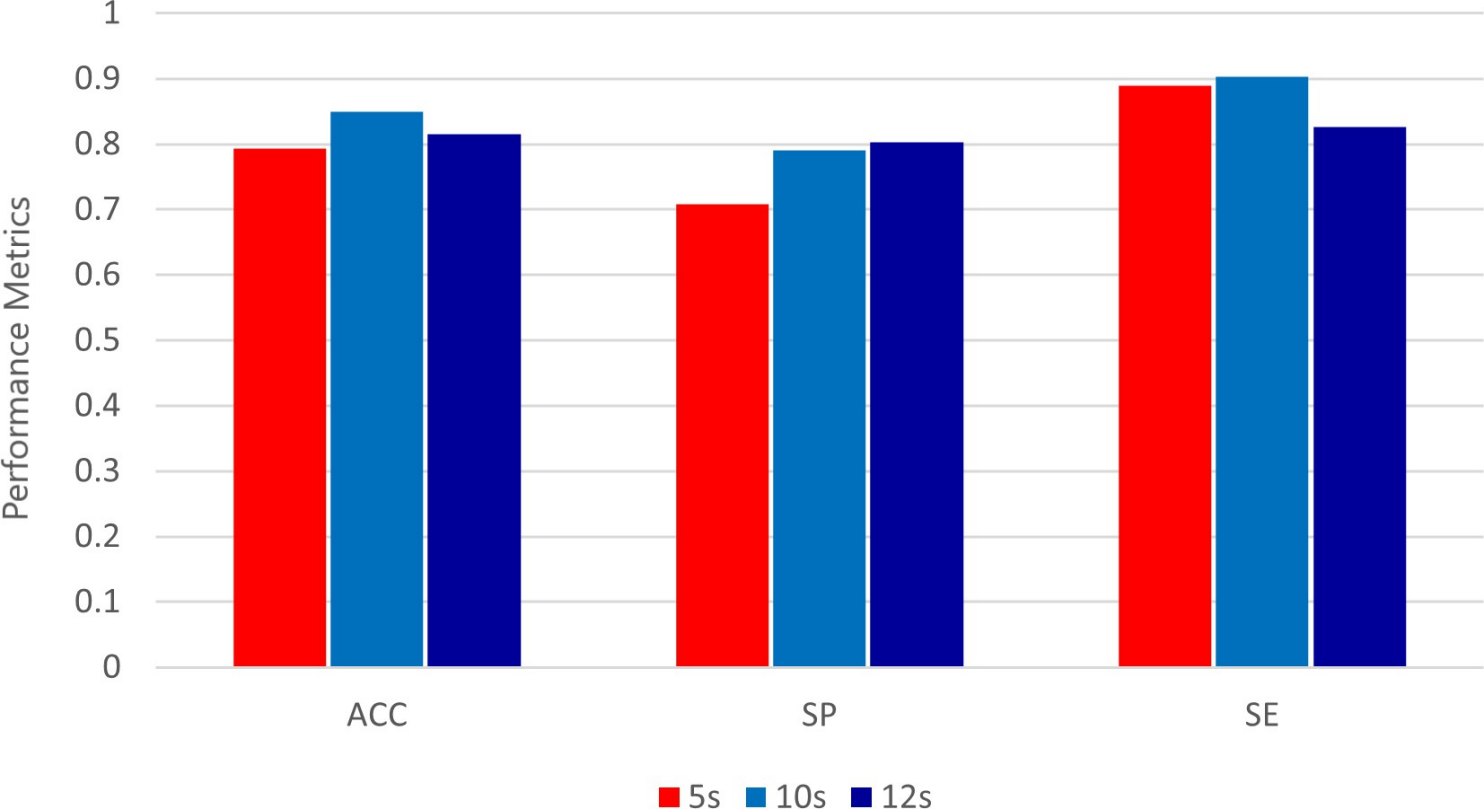
Accuracy, specificity and sensitivity of three classification test sets.

In our classification research, we carefully evaluate the performance of the classifier on a series of different test sets, and each test set is characterized by different sample sizes: 4800, 2400 and 2000 samples respectively. This comprehensive analysis is intuitively shown in Fig 9.

As revealed by the discrimination results described therein, the classifier shows its best performance in a time window of 10 seconds. This highest performance is reflected in the accuracy and sensitivity of all three test sets, which shows its strength in the field of diagnostic accuracy. In addition, when evaluating the specificity, compared with other time windows, the classifier still reaches the peak of its discrimination ability, except for the 12-second window, in which it shows a noteworthy exception.

This performance test not only emphasizes the proficiency of the classifier but also provides valuable insights into the best time window to improve the accuracy and sensitivity of our classification tasks.

Table 3 shows the confusion matrix of the test data. The system aims to predict whether the EEG at this time is in a motion sickness state. The test data consists of 2400 EEG pattern records. The test set includes 1257 signal features in the normal case and 1143 signal features in the case of motion sickness. Based on the confusion matrix, the system incorrectly predicts the real categories with 197 patterns, with a false positive rate of about 8.2%. For this study, most classifiers can identify whether this segment of EEG belongs to the real state obtained by motion sickness.

**Table 3:** Confusion matrix of 2400 samples.

	Real state	
**Prediction**	Normal	Sickness
**Normal**	1134	238
**Sickness**	123	905

**Fig 10:**
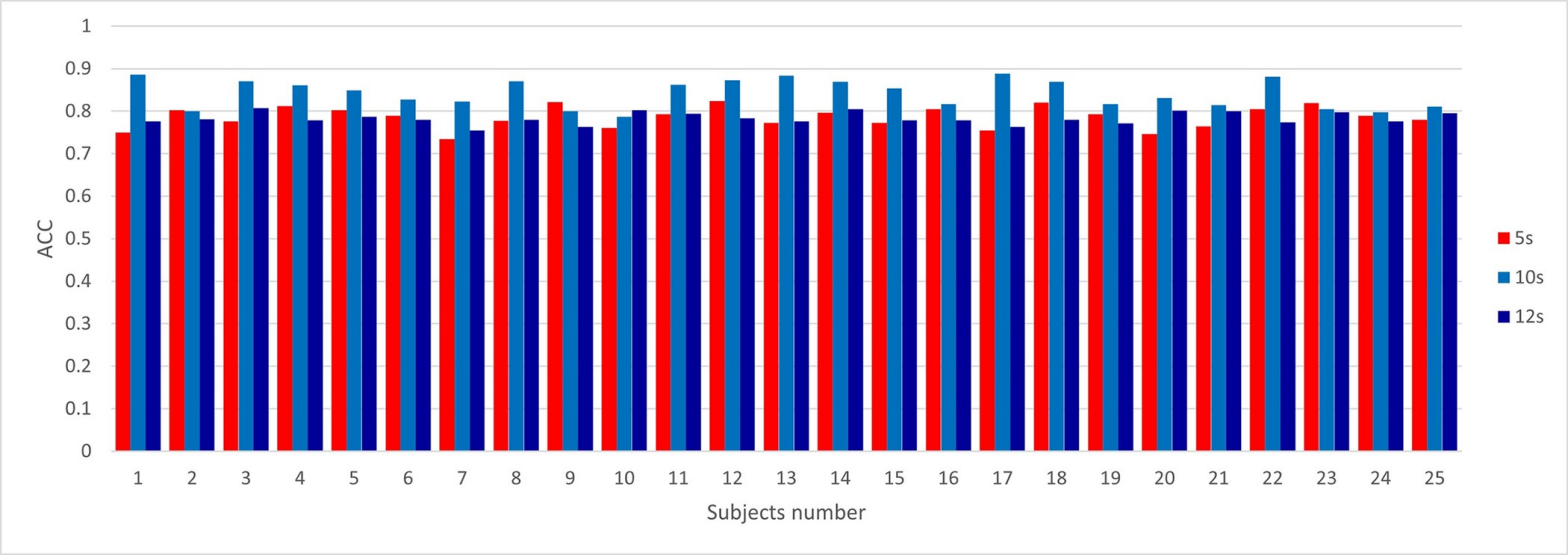
The classification accuracy performance of the classifier on the segmented EEG data set of each experimental object.

In Fig 10, By using EEG classification data sets with different lengths, we tested a single individual on the classifier and achieved remarkable classification results. In the test process, we use EEG signals with different lengths as input, which verifies the robustness of the classifier when dealing with data with different time spans. Encouragingly, we observed that the classification accuracy of a single individual is mostly between 0.75 and 0.9, which shows that our classifier can effectively capture the EEG patterns of individuals in different time scales and achieve high classification performance. This discovery is of great significance to the personalized application of brain-computer interfaces and the in-depth understanding of individual differences.

Table 4 shows the performance test of comparing the machine learning algorithm with our proposed classifier.

**Table 4:** Performance comparison with other machine learning models.

		ACC	SP	SE
**Voting Classifier**		0.763	0.719	0.801
**Polynomial-SVM**		0.792	0.778	0.782
**Proposed Classifier**	**5s**	0.793	0.708	0.890
	**10s**	***0*.*849***	***0*.*791***	***0*.*903***
	**12s**	0.815	0.803	0.826

Firstly, judging from the performance of two existing classifiers (Voting Classifier and Polynomial-SVM) [44], Polynomial-SVM is slightly better than Voting Classifier in accuracy and precision. However, the Voting Classifier performs better in sensitivity. This shows that the Voting Classifier is more sensitive in identifying positive categories (positive), while Polynomial-SVM is better in overall accuracy and accuracy.

Secondly, for the proposed classifier, we can observe that different time intervals (5 seconds, 10 seconds, 12 seconds) have a significant impact on the performance index. In all three cases, the accuracy is between 0.793 and 0.849, while the accuracy varies from 0.708 to 0.791. However, the sensitivity varies at different time intervals, ranging from 0.826 to 0.903. This shows that the proposed classifier has different sensitivity in identifying positive categories at different time intervals.

Finally, we need to pay special attention to the excellent performance of the proposed classifier in the 10-second time interval, with the highest accuracy (0.849) and accuracy (0.791) and high sensitivity (0.903). This means that within a 10-second time interval, the classifier performs well in correct classification and high accuracy, and is more sensitive to the detection of positive categories.

To sum up, we can conclude that the proposed classifier shows different performances at different time intervals, among which the classifier with a 10-second time interval shows the best performance with high accuracy, precision, and sensitivity. This means that in the corresponding application scenario, it is very important to choose the appropriate time interval to obtain the best performance.

### 3.2 Regression prediction results

According to the previous description, for the predictor, we trained three prediction models by using 5s, 10s, and 20s as the length of the prediction window respectively. In the same 50 iterations, the model loss of 20s prediction window has been reduced to less than 0.2, while the model loss of 5s prediction window meets the requirements, but its prediction effect is not ideal, so we finally choose 10s prediction window model as the best prediction window of the predictor.

**Fig 11:**
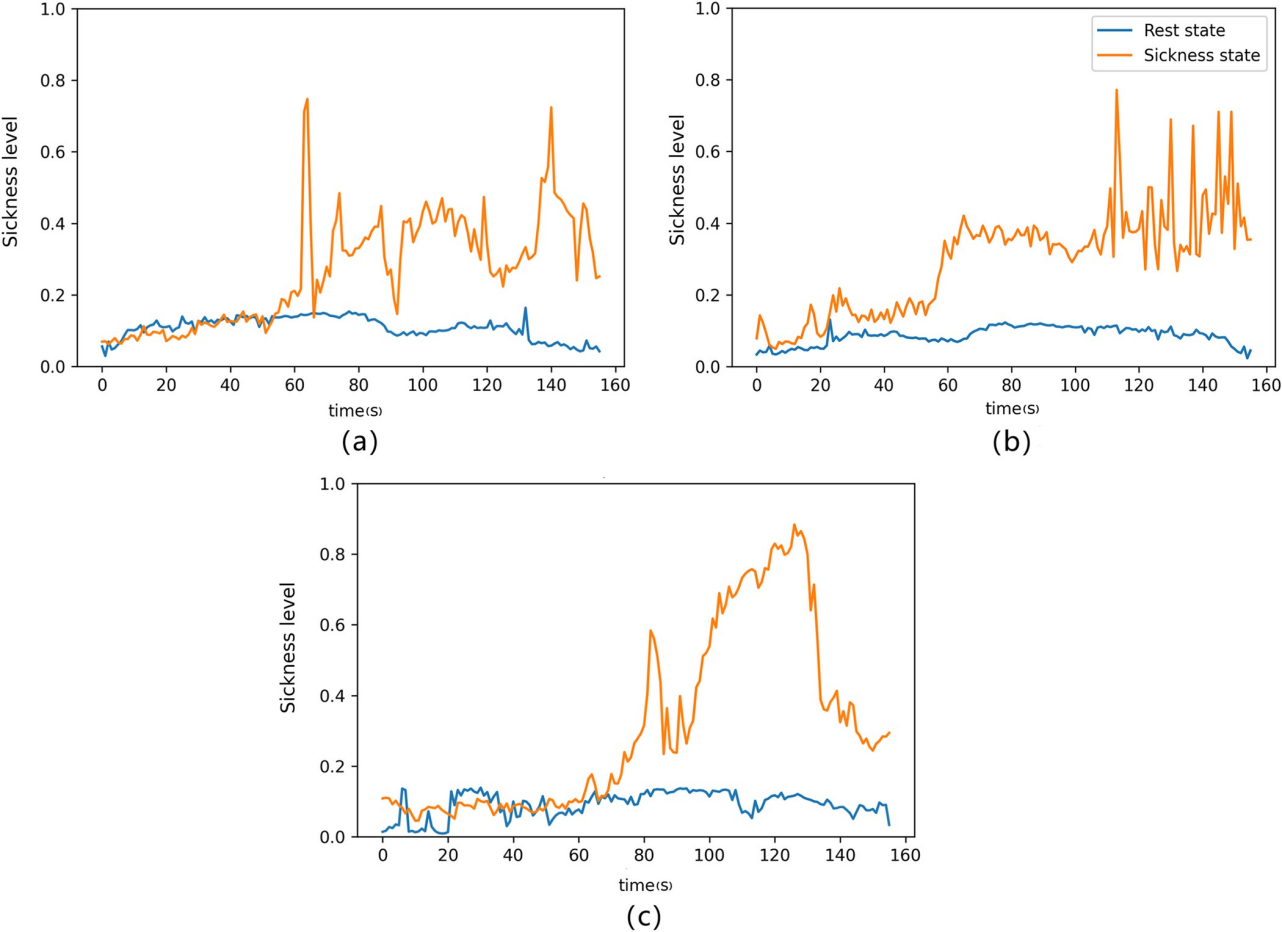
Real-time prediction effect of 5s(a),10s(b),20s(c) prediction window, the blue curve represents rest, and orange represents task state.

Fig 11 vividly illustrates the predictor’s performance in forecasting motion sickness levels during both the resting and task states. Although the model uses three different prediction lengths, the experimenter’s EEG began to show motion sickness in about 1 minute, and then rose for a period of time, maintaining a certain level of motion sickness. After that, the prediction effect of using 5s and 20s windows was to keep this state until the end, while the prediction effect of using 10s window was a jumping state.

The real-time motion sickness state of the experimental subjects is perceptible, showcasing a rapid increase in motion sickness level at approximately 56 seconds. Subsequently, the motion sickness level fluctuates continuously for about 1 minute and 56 seconds. It is important to acknowledge that while our proposed predictor is highly accurate, it may not always precisely match the real-time motion sickness level. This divergence might be attributed to potential delays in the subjects’ reporting of their scores. In future research endeavors, we plan to implement a real-time motion sickness level recording system to facilitate instantaneous and precise data recording. This improvement will enhance the alignment between our predictor’s predictions and the real-time motion sickness levels.

**Fig 12:**
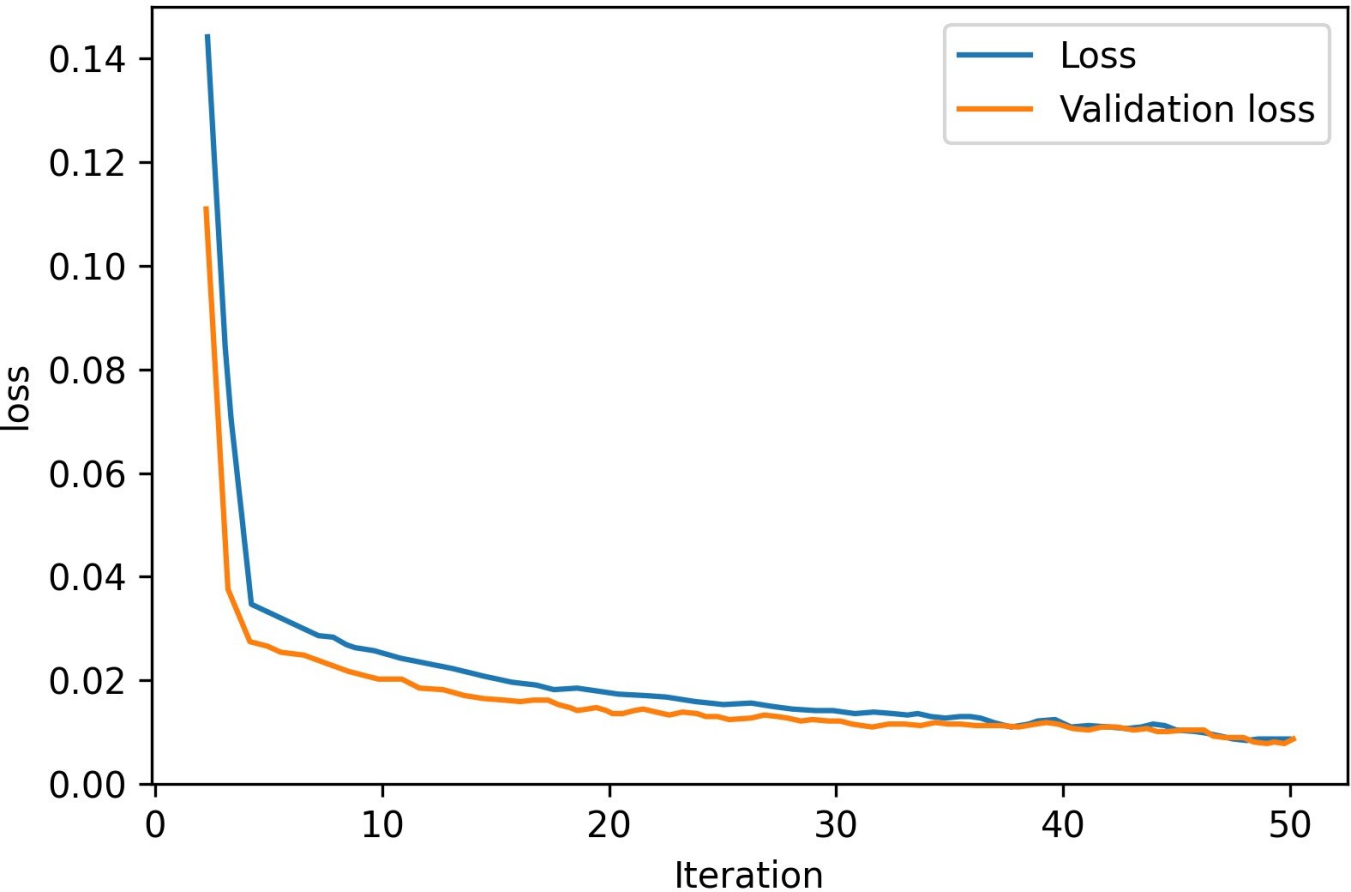
Loss curve of predictor training.

Fig 12 presents the Loss curve of predictor training process; it is evident that the loss consistently diminishes to a remarkable low of 0.0078 after 50 iterations on the test dataset.

We also compared the results with the most advanced methods. Table 5 shows the comparison results between these algorithms. The model will be evaluated with the lower baseline.

**Table 5:** Performance comparison between our predictor and other methods.

Objective Methods	PLCC	SROCC	RMSE
**Heart Rate**	0.215	0.217	21.172
**FUSION NET**	0.854	0.700	17.877
**VRSA NET**	0.885	0.882	10.251
**Proposed Predictor**	***0*.*904***	***0*.*915***	***8*.*872***

According to the performance index in superscript, we focus on the comparison between our Proposed Predictor and other methods [45], which is helpful to highlight the superiority of our model. Firstly, it is observed that Heart Rate, as an objective method, is relatively poor in emotional state recognition. Its PLCC and SROCC are only 0.215 and 0.217 respectively, and the RMSE is as high as 21.172, which means that its prediction accuracy is low and there are significant errors. Then, FUSION NET showed better performance than heart rate, and its PLCC and SROCC reached 0.854 and 0.700 respectively. However, its RMSE is still relatively high, reaching 17.877, suggesting that there is still a certain degree of error. It is further observed that the performance of VRSA NET is better than the first two, with higher PLCC and SROCC, reaching 0.885 and 0.882 respectively. In addition, RMSE is significantly reduced to 10.251, indicating that it has higher prediction accuracy and reliability.

Crucially, our Proposed Predictor performs well in all performance indicators, with PLCC and SROCC as high as 0.904 and 0.915 respectively, while RMSE is only 8.872. This means that our model has higher accuracy and lower error in the task of emotional state recognition, and has obvious advantages compared with other methods.

To sum up, our prediction model is superior to other methods in objective emotional state recognition, and provides a more accurate and reliable solution for this field.

## 4. Conclusion

This paper introduces an innovative deep-learning model founded on cyclic neural networks. It excels in classifying motion sickness states within specific time frames and real-time prediction across varied window lengths. Our experiments showcase the model’s superiority over conventional machine learning methods and recent deep learning models. Notably, both the predictor and classifier demonstrate remarkable performance improvements, showcasing enhanced accuracy in signal prediction across different window lengths and improved recognition in diverse sample set sizes.

Moving forward, our research aims to expand the sample size, addressing survivor bias concerns. Additionally, there’s a focus on enhancing the accuracy and reliability of motion sickness prediction algorithms grounded in EEG data, emphasizing standardization for comparative assessments of model performance [46].

Future endeavors will integrate eye movement data, posture swing information, and EEG data to craft a multi-modal prediction and evaluation model. The objective is to significantly boost prediction accuracy. Furthermore, incorporating real-time feedback from participants aims to delve deeper into the correlation between predictions and actual occurrences. This comprehensive analysis seeks to unveil the relationship between motion sickness prediction and real-time manifestation, ultimately striving to preemptively predict and mitigate motion sickness for VR users.
